# Increased high-risk plaque burden in type 2 diabetes: a 10-year follow-up study

**DOI:** 10.1186/s12933-025-02977-1

**Published:** 2025-11-05

**Authors:** Emilie L. Gaillard, Sophie H. M. Cramer, Nordin M. J. Hanssen, Michiel J. Bom, Steven A. J. Chamuleau, R. Nils Planken, Andrew D. Choi, S. Matthijs Boekholdt, Erik S. G. Stroes, Paul Knaapen, Nick S. Nurmohamed

**Affiliations:** 1 Department of Cardiology, Amsterdam UMC, Vrije Universiteit Amsterdam, Amsterdam, The Netherlands; 2 Department of Vascular Medicine, Amsterdam UMC, University of Amsterdam, Amsterdam, The Netherlands; 3 Division of Cardiology, The George Washington University School of Medicine, Washington, DC USA; 4 Department of Radiology and Nuclear Medicine, Amsterdam UMC, Universiteit Van Amsterdam, Amsterdam, The Netherlands; 5 Department of Radiology, Mayo Clinic, Rochester, USA

**Keywords:** Type 2 diabetes mellitus, Coronary computed tomography angiography, Coronary artery disease, Atherosclerosis, Coronary plaque burden, Coronary plaque composition, Coronary plaque progression, Vulnerable plaque

## Abstract

**Background:**

Using serial coronary CT angiography (CCTA) imaging, we aimed to characterize baseline coronary plaque characteristics and quantify 10-year coronary plaque progression, including high-risk and low-density plaque presence, in patients with and without type 2 diabetes.

**Methods:**

A total of 299 patients underwent CCTA with a median scan interval of 10.2 [IQR 8.7–11.2] years. Patients who underwent coronary artery bypass grafting and vessels revascularized by percutaneous coronary intervention were excluded (n = 32). Scans were analyzed using atherosclerosis imaging-quantitative CCTA analysis (AI-QCT; Cleerly Inc.). Associations between diabetic status, baseline and follow-up plaque burden and characteristics were evaluated using multivariable regression adjusted for cardiovascular risk factors, statin use, baseline plaque volumes, and scanner settings.

**Results:**

In total, 267 patients were included (mean age 57 ± 7 years; 43% were women), 44 (16.5%) had type 2 diabetes (HbA1c 56 ± 14 mmol/mol). At baseline, patients with diabetes had a higher percent atheroma volume (PAV) compared to non-diabetic individuals (5.1% [1.7, 10.9] versus 2.2% [0.5, 5.8]). Adjusted for cardiovascular risk factors, patients with diabetes had a higher plaque burden at both baseline and follow-up. After adjustment for cardiovascular risk factors and baseline plaque volumes, individuals with diabetes had a more than threefold higher rate of plaque progression. After 10 years of follow-up, patients with diabetes had a higher prevalence of both high-risk plaque (OR 2.75; 95% CI 1.38–5.48; *p* = 0.004) and low-density plaque (OR 2.88; 95% CI 1.45–5.70; *p* = 0.002).

**Conclusions:**

Patients with diabetes had a more than threefold higher rate of coronary plaque progression and an increased development of high-risk plaque.

**Graphical abstract:**

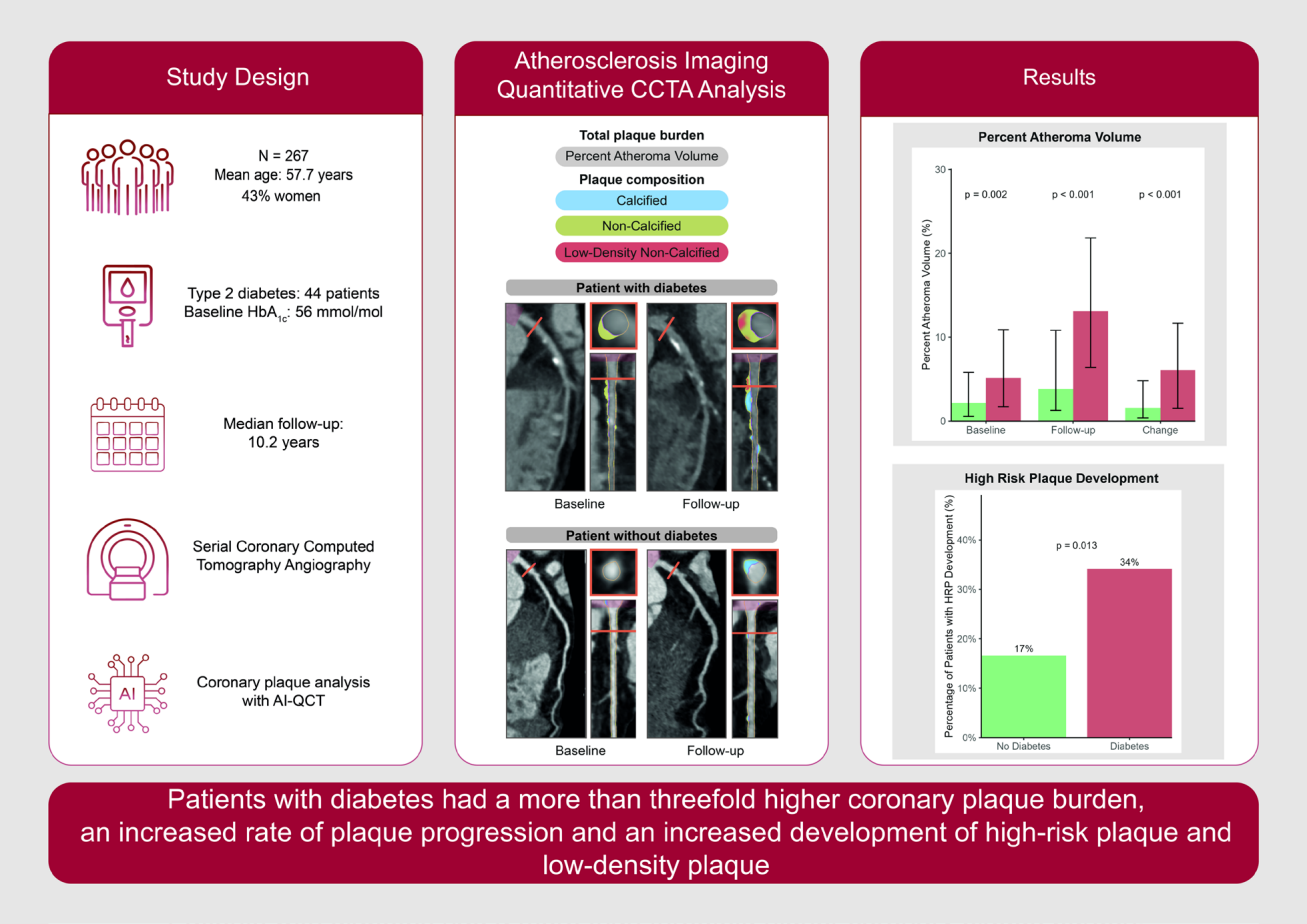

**Supplementary Information:**

The online version contains supplementary material available at 10.1186/s12933-025-02977-1.

## Research insights

**What is currently known about this topic?**

- Patients with type 2 diabetes have increased cardiovascular risk.
- Data on long-term coronary plaque progression and high-risk plaque development is limited.

**What is the key research question?**

- How does type 2 diabetes affect long-term coronary plaque progression and high-risk phenotypes during long-term follow-up?

**What is new?**

- Patients with type 2 diabetes demonstrated a twofold higher baseline coronary plaque burden.
- Patients with type 2 diabetes had more than threefold greater plaque progression rates over the long-term follow-up of 10 years.
- Patients with type 2 diabetes showed increased prevalence and development of high-risk plaque.

## How might this study influence clinical practice?

- Findings support intensive cardiovascular risk management in patients with type 2 diabetes.

## Background

Atherosclerotic cardiovascular disease (ASCVD) is the leading cause of morbidity and mortality in patients with type 2 diabetes [1, 2]. Individuals with type 2 diabetes are characterized by a combination of cardiometabolic risk factors, including hyperglycemia, dyslipidemia, obesity, systemic inflammation and hypertension; all of which have been implicated to contribute to a pro-atherogenic state [3]. Despite contemporaneous medication targeting these traditional cardiovascular risk factors, type 2 diabetic patients are still hallmarked by a residual ASCVD risk [4]. The quest for further optimization of ASCVD prevention in type 2 diabetes has therefore re-invigorated the question whether and to what extent diabetes itself, independent of these other cardiometabolic risk factors, contributes to coronary plaque progression and concomitant plaque instability.

Experimentally, injections causing transient hyperglycemia in mice were found to potently increase atherogenesis progression [5]. In type 2 diabetic patients, a recent cross-sectional study demonstrated that the prevalence of atherosclerosis measured with coronary computed tomographic angiography (CCTA) increased with worsening of glycemic control [6]. Similarly, a recent longitudinal study demonstrated that the combination of hyperglycemia and low high density lipoprotein cholesterol (HDL-C) independently predicted rapid plaque progression [7]. Intracoronary imaging studies using optical coherence tomography also reported phenotypical changes with higher macrophage density in the coronary arteries of type 2 diabetic patients compared with non-diabetic patients [8]. However, long-term follow-up studies describing both plaque progression and plaque phenotypical changes in type 2 diabetic patients are lacking.

CCTA has evolved into an effective non-invasive imaging modality for detecting coronary artery disease (CAD), allowing for reproducible quantification of total plaque burden as well as reliable determination of plaque composition. CCTA studies have helped to elucidate the process of atherosclerotic disease progression and have identified high risk plaque phenotypes with prognostic value for future cardiac events [9, 10]. Over the past years, artificial intelligence-guided analysis (AI) has increased accuracy and reproducibility of atherosclerosis quantification with CCTA demonstrating excellent performance [11, 12]. Cross-sectional CCTA studies in type 2 diabetes patients reported a greater overall plaque burden [13]. Additional studies have shown increased low-density and calcified plaque volumes in type 2 diabetic patients compared with healthy individuals [14, 15].

We hypothesized that individuals with type 2 diabetes experience more rapid plaque progression and a predisposition to the development of adverse plaque phenotypes, compared with individuals without type 2 diabetes. We tested this hypothesis by conducting a serial CCTA study, analyzed with state-of-the-art AI-guided techniques, in patients with and without type 2 diabetes to investigate the impact of type 2 diabetes on long-term plaque progression and change in plaque phenotype.

## Methods

### Study design and population

This prospective long-term serial CCTA study was conducted in a cohort of patients at the Amsterdam University Medical Centers. Patients underwent baseline CCTA imaging for suspected CAD between 2008 and 2014. At baseline, patients had no history of CAD. The follow-up study was separately approved by the local ethics committee [16]. The study complied with the Declaration of Helsinki. All participants provided written informed consent. Out of 465 patients considered for follow-up imaging, 299 patients consented to a follow-up CCTA. Of these, 61 patients underwent percutaneous coronary intervention (PCI) and 32 patients underwent coronary artery bypass grafting between the baseline and follow-up CCTA, with 3 patients receiving both procedures. The 32 patients undergoing coronary artery bypass grafting were excluded from the present analysis, resulting in a final study population of 267 patients. Diabetes was defined based on documented medical history and patient self-report at baseline and follow-up.

### CCTA imaging

At baseline, all patients underwent CCTA using ≥ 64 slice CCTA scanners (Philips Healthcare, Best, the Netherlands) [17, 18]. Patients were administered sublingual nitroglycerin and metoprolol, if necessary, to attain a heart rate below 65 beats per minute. At baseline, coronary artery calcium scoring (CACS) was initially obtained, followed by contrast-enhanced CCTA using 120 kV tube voltage with prospective electrocardiogram gating triggered at 75% of the R-R interval. Contrast administration consisted of 100 ml iobitridol (Xenetix 350, Guerbet Nederland B.V., Gorinchem, the Netherlands) with automated bolus tracking. At follow-up, CCTA and CACS were also performed using a third‐generation dual source CT scanner (SOMATOM Force, Siemens Healthineers, Germany) with automated tube voltage and tube current modulation (CAREKv, CAREDose 4D, Siemens Healthineers, Germany). Patients received identical pre-medication protocols. CCTA was acquired using prospective electrocardiogram gating triggered at 70% of the R-R interval, with weight and kV-adjusted contrast dosing (Xenetix 350) following test bolus determination. Scanner settings and protocols differed between baseline and follow-up scans. We adjusted for kV and scanner type at follow-up in our statistical analyses.

### Artificial intelligence-guided CCTA analysis

CCTA images underwent AI-guided analysis using Atherosclerosis Imaging Quantitative Computed Tomography (AI-QCT) software (Cleerly Inc., Denver, CO). The accuracy and reproducibility of the AI-QCT algorithm have been described previously [11]. Coronary segments with a vessel diameter ≥ 1.5 mm were included in the analysis according to the modified 18-segment Society of Cardiovascular Computed Tomography (SCCT) model. Coronary atherosclerosis was defined as any tissue structure exceeding 1 mm^2^ within the coronary artery wall that could be distinguished from surrounding epicardial tissue, epicardial fat, and the vessel lumen itself. Coronary plaque volumes were adjusted for differences in coronary artery size by normalizing it to the vessel volume, calculated as plaque volume in mm^3^ divided by vessel volume in mm^3^ and multiplied by 100% on a per-patient level. These adjusted volumes were reported as percent atheroma volume (PAV), percent calcified plaque volume (CPV) defined as > 350 HU and percent non-calcified plaque volume (NCPV) defined as HU between 30 and 350. The presence of low-density non-calcified plaque (< 30 Hounsfield Unit (HU)) was evaluated, as well as high-risk plaque features, which were characterized by the presence of positive remodeling and low attenuation plaque. A patient was classified as having high-risk plaque if at least one coronary lesion demonstrated both positive remodeling and low-density components simultaneously. High-risk plaque (HRP) and low-density plaque (LDP) development were defined as the incident appearance of plaque features during follow-up in patients without those features at baseline. Development rates were calculated as the percentage of at-risk patients who developed the feature during follow-up.

For the serial analysis, vessels with impaired image quality due to motion artifacts, inadequate opacification, beam hardening, or other technical issues were excluded from both baseline and follow-up assessments. Coronary arteries with stents at follow-up were excluded from analysis at both time points to maintain consistency in 1-to-1 comparisons. In total, 10.5% of coronary vessels were excluded because of poor image quality or stent placement. A vessel-by-vessel matched analysis was performed, comparing identical coronary vessel trajectories at baseline and follow-up scans to ensure accurate assessment of plaque progression.

### Statistical analysis

The absolute change in coronary plaque volumes was calculated by subtracting baseline values from follow-up values. The relationship between type 2 diabetes and plaque volumes over time was assessed using linear regression models. The multivariable models were adjusted for age, sex, and cardiovascular risk factors (body mass index (BMI), systolic blood pressure, low density lipoprotein cholesterol (LDL-C), lipoprotein(a) (Lp[a]), triglycerides, hypertension, smoking and family history of premature CAD, statin treatment, scanner settings and baseline plaque volumes. Data are presented as mean ± standard deviation for normally distributed variables or median with interquartile range (IQR) for non-normally distributed data. Categorical variables are expressed as absolute numbers and percentages. Independent sample t-tests, Wilcoxon tests, Mann–Whitney U-tests and Kruskal–Wallis tests were used where appropriate. All statistical analyses were performed using RStudio software version 4.3.2 (R Foundation, Vienna, Austria).

## Results

### Patient characteristics

Table 1 lists the baseline characteristics for the study cohort, comprising 267 patients, 44 (16%) of whom had type 2 diabetes. At baseline, patients had a mean age of 57 ± 7 years and 153 (57%) were male. Sex distribution and age were similar between groups. BMI was higher in the individuals with diabetes (29.8 ± 4.6 vs. 26.3 ± 4.0 kg/m^2^; *p* < 0.001). Cardiovascular risk factors including hypertension (59% vs. 38%; *p* = 0.010) and hypercholesterolemia (61% vs. 32%; *p* < 0.001) were more prevalent among those with diabetes. LDL-C levels (2.3± 0.9 vs. 2.7 ± 1.0 mmol/l; *p* = 0.010) were lower in diabetic patients, consistent with a higher proportion of diabetic patients using statins. Cardiovascular risk factor management, including smoking status and systolic blood pressure control, was similar between patients with and without diabetes. Coronary atherosclerosis severity of the baseline CCTA, assessed using Coronary Artery Disease—Reporting and Data System (CAD-RADS), showed no significant differences between groups. In diabetic patients, median HbA1c was 56 ± 14 mmol/mol, whereas albuminuria was present in 16 individuals (28%). At follow-up, 3 patients developed de novo diabetes, resulting in a total of 47 individuals with diabetes (18%).

**Table 1:** Baseline characteristics

Characteristic	Overall N = 267	Diabetes N = 44	No diabetes N = 223	*p*-value
Age (years), mean (SD)	57 ± 7.3	58 ± 7.4	57 ± 7.3	0.337
Male sex	153 (57%)	27 (61%)	126 (57%)	0.551
BMI (kg/m^2^)	26.9 ± 4.3	29.8 ± 4.6	26.3 ± 4.0	< 0.001
Hypertension	111 (42%)	26 (59%)	85 (38%)	0.010
Systolic blood pressure (mmHg)	138 ± 20	141 ± 19	137 ± 20	0.202
Hypercholesterolemia	99 (37%)	27 (61%)	72 (32%)	< 0.001
Smoking history	80 (30%)	9 (20%)	71 (32%)	0.132
Family history of premature CAD	148 (55%)	20 (45%)	128 (57%)	0.145
LDL cholesterol (mmol/l)	2.6 ± 1.0	2.3 ± 0.9	2.7 ± 1.0	0.010
HDL cholesterol (mmol/l)	1.4 ± 0.5	1.2 ± 0.4	1.5 ± 0.6	< 0.001
Triglycerides (mmol/l)	1.4 (0.9, 2.1)	1.8 (1.2, 2.4)	1.3 (0.9, 2.0)	0.004
Lipoprotein(a) (nmol/l)	25 (8, 111)	23 (8, 73)	25 (8, 113)	0.372
eGFR (ml/min/1.73m^2^)	97 (83, 112)	107 (88, 126)	95 (82, 110)	0.021
Albuminuria				
No	–	28 (72%)	–	
Micro	–	9 (23%)	–	
Macro	–	2 (5.1%)	–	
HbA1c (mmol/mol)	–	56 ± 14	–	
Duration of diabetes (years)	–	7.5 ± 7.4	–	
Metformin	–	30 (70%)	–	
Insulin	–	13 (30%)	–	
Statin use				0.201
No	102 (38%)	11 (25%)	91 (41%)	
Low intensity	3 (1.1%)	0 (0%)	3 (1.3%)	
Moderate intensity	133 (50%)	27 (61%)	106 (48%)	
High intensity	29 (11%)	6 (14%)	23 (10%)	
Beta-blocker use	154 (59%)	26 (62%)	128 (58%)	0.631
Aspirin use	185 (70%)	29 (69%)	156 (71%)	0.841
Use of calcium antagonists	62 (24%)	9 (21%)	53 (24%)	0.721
CAD-RADS				0.734
0	10 (3.7%)	1 (2.3%)	9 (4.0%)	
1	135 (51%)	19 (43%)	116 (52%)	
2	45 (17%)	9 (20%)	36 (16%)	
3	35 (13%)	6 (14%)	29 (13%)	
4/5	42 (16%)	9 (20%)	33 (15%)	
Coronary artery calcium score	28 (0, 231)	143 (4, 396)	23 (0, 189)	0.011

Data are presented as mean (SD), median (quartile 1, quartile 3) or n (%). Abbreviations: BMI, body mass index (calculated as weight in kilograms divided by height in meters squared); CAD, coronary artery disease; CAD-RADS, coronary artery disease reporting and data system; eGFR, estimated glomerular filtration; HbA1c, Hemoglobin A1c; HDL, high-density lipoprotein; LDL, low-density lipoprotein

### Baseline plaque characteristics

**Fig. 1:**
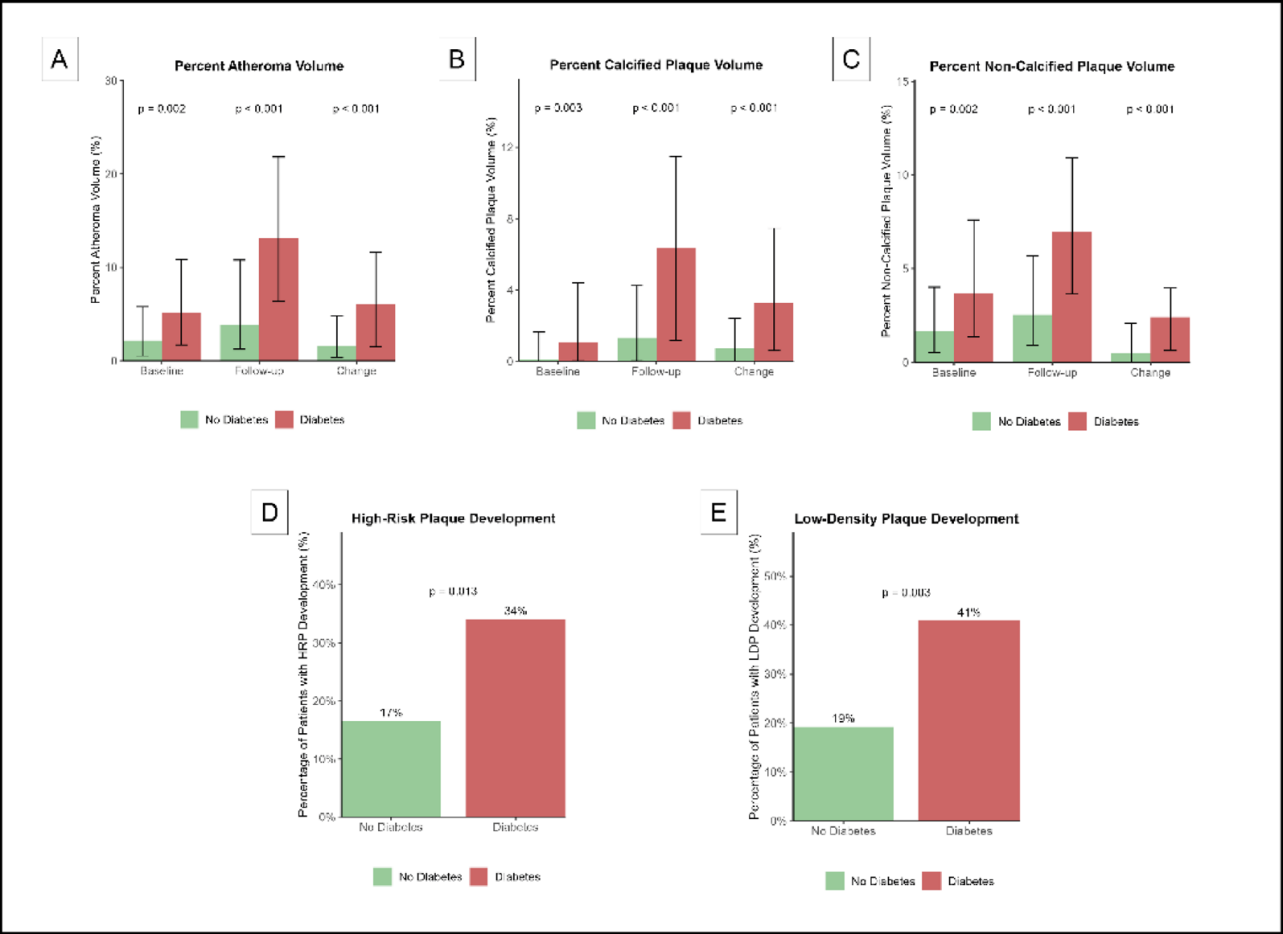
Coronary plaque characteristics in patients with and without type 2 diabetes. Bar charts showing plaque measurements at baseline, follow-up, and change (**A**–**C**) or development of plaque after 10-year follow up (**D**–**E**) by diabetes status. **A** Percent Atheroma Volume (PAV), **B** Percent Calcified Plaque Volume, and **C** Percent Non-Calcified Plaque Volume show median values with interquartile ranges. **D** High-Risk Plaque (HRP) and **E** Low-Density Plaque (LDP) show the percentage of patients who developed new-onset HRP or LDP during follow-up, respectively. Green bars: no diabetes; red bars: diabetes. P-values from Wilcoxon rank-sum tests (**A**–**C**) or chi-square tests (**D**–**E**)

Individuals with diabetes had a higher percent atheroma volume at baseline compared to non-diabetic individuals (5.1% vs 2.2%; *p* = 0.02) (Fig. 1A). With respect to plaque composition, percent non-calcified plaque volume was higher in individuals with diabetes at baseline (3.7% vs 1.7%; *p* = 0.02) (Fig. 1C). Similarly, percent calcified plaque volume was elevated in the diabetic group (1.1% vs 0.1%; *p* = 0.003) (Fig. 1B). At baseline, diabetic patients did not exhibit a higher prevalence of low-density (14% vs 15%; *p* = 0.786) or high-risk plaques (43% vs 30%; *p* = 0.089) (Supplementary Table 2).

### Coronary plaque progression

**Fig. 2:**
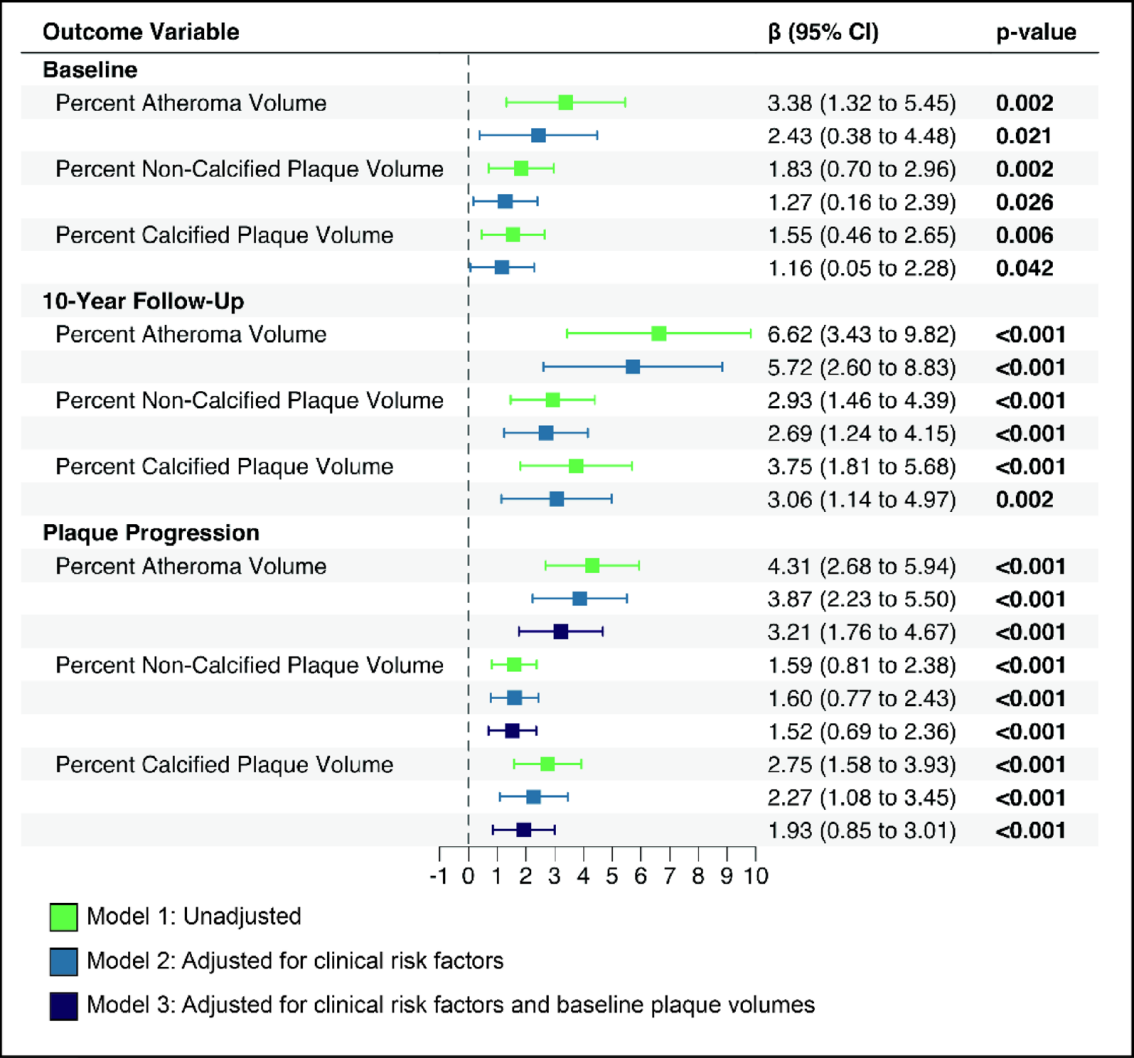
Association between type 2 diabetes and coronary plaque burden and progression. Beta coefficients comparing patients with type 2 diabetes to non-diabetic controls from linear regression models. Model 1: unadjusted; Model 2: adjusted for demographics (age, sex) and cardiovascular risk factors (BMI, systolic blood pressure, LDL cholesterol, lipoprotein(a), triglycerides, hypertension, current smoking, family history of premature coronary artery disease) and statin use; Model 3: additionally adjusted for baseline plaque volumes

Over the 10-year follow-up period, patients with diabetes had a nearly fourfold greater progression of percent atheroma volume (6.0% vs 1.6%; *p* < 0.001) (Fig. 1A). Diabetic patients demonstrated greater calcified plaque (3.3% vs 0.7%; *p* < 0.001) and non-calcified plaque progression (2.4% vs 0.5%; *p* < 0.001) (Fig. 1B, C). To further elucidate the effect of diabetes on coronary plaque progression, a linear regression analysis was conducted. In the univariate analysis, diabetes was associated with 4.31 percent atheroma progression (β = 4.31, 95% CI 2.68–5.94; *p* < 0.001), 1.59 percent non-calcified plaque volume progression (β = 1.59, 95% CI 0.81–2.38; p < 0.001), and 2.75 percent calcified plaque (β = 2.75, 95% CI 1.58–3.93; *p* < 0.001) progression (Fig. 2). In the multivariate linear regression model, after adjustment for sex, age, cardiovascular risk factors (BMI, systolic blood pressure, LDL-C, Lp(a), triglycerides, smoking and family history of premature CAD), statin treatment and scanner settings, diabetes remained positively associated with 3.87 percent atheroma progression (β = 3.87, 95% CI 2.23–5.50; *p* < 0.001), 1.60 percent non-calcified plaque volume progression (β = 1.60, 95% CI 0.77–2.43 *p* < 0.001) and 2.27 percent calcified plaque volume progression (β = 2.27, 95% CI 1.08–3.45; p < 0.001) (Fig. 2). After further adjustment for baseline plaque volumes, diabetes still remained associated with increased coronary plaque volume progression in all plaque volumes; 3.21 percent atheroma volume progression (β = 3.21, 95% CI 1.76–4.67; *p* < 0.001), 1.52 percent non-calcified plaque volume progression (β = 1.52, 95% CI 0.69–2.36; *p* < 0.001) and 1.93 percent calcified plaque volume progression (β = 1.93, 95% CI 0.85–3.01; *p* < 0.001) (Fig. 2).

### High-risk plaque development

At the patient level, individuals with diabetes demonstrated significantly higher rates of high-risk plaque development (34% vs. 17%, *p* = 0.013) and low-density plaque development (41% vs. 19%, *p* = 0.003) compared to those without diabetes (Fig. 1D, E). After adjustment for baseline percent atheroma volume, diabetes was independently associated with an increased risk of developing high-risk plaques (adjusted OR: 2.57, 95% CI: 1.21–5.44, *p* = 0.014) and low-density plaques (adjusted OR: 2.69, 95% CI 1.32–5.47, *p* = 0.006; Table 2). Similarly, at follow-up imaging, diabetic patients exhibited significantly more low-density plaques (39% vs 17%; p = 0.002) and high-risk plaques (64% vs 36%; *p* < 0.001) (Supplementary Table 2). Diabetes was associated with an odds ratio (OR) of 2.75 (95% CI, 1.38–5.48; *p* = 0.004) for the presence of high-risk plaque and an OR of 2.88 (95% CI, 1.45 -5.70; *p* = 0.002) for the presence of low-density plaque at follow-up (Supplementary Table 3). After adjustment for baseline percent atheroma volume, diabetes was associated with an OR of 2.33 (95% CI, 1.11–4.88; *p* = 0.025) and 2.50 (95% CI, 1.21–5.17 *p* = 0.014) for the presence of high-risk plaque and low-density plaque, respectively (Supplementary Table 3).

**Table 2:** Association between type 2 diabetes and coronary plaque development

Outcome	Model	OR (95% CI)	*p*-value
High-risk plaque development	1	2.55 (1.21–5.34)	0.013
	2	2.57 (1.21–5.44)	0.014
	3	2.44 (1.03–5.78)	0.042
Low-density plaque development	1	2.84 (1.41–5.73)	0.004
	2	2.69 (1.32–5.47)	0.006
	3	3.61 (1.56–8.39)	0.003

Adjusted odds ratios from logistic regression models for adverse coronary plaque development in patients with type 2 diabetes. Model 1: Unadjusted analysis. Model 2: Adjusted for baseline percent atheroma volume. Model 3: Adjusted for baseline percent atheroma volume, demographics (sex, age) and cardiovascular risk factors (BMI, systolic blood pressure, LDL cholesterol, lipoprotein(a), triglycerides, hypertension, current smoking, family history of premature coronary artery disease) and statin use

## Discussion

Here, we show that individuals with type 2 diabetes have a more than threefold higher coronary plaque progression rate during 10-year follow-up compared with non-diabetic controls, which persisted after adjustment for traditional cardiovascular risk factors and baseline plaque volume. Moreover, a higher propensity towards the development of adverse plaque features was observed in diabetic patients, even after adjustment for baseline plaque volumes. Collectively, these data confirm the significant impact of diabetes on coronary plaque progression and the development of adverse plaque phenotypes, despite similar control of traditional cardiovascular risk factors.

### Plaque progression

Whereas the higher prevalence of calcified plaque in diabetes was reported previously, we show an approximately threefold higher 10-year progression rate of both total plaque volume and of non-calcified plaque volume [14, 15, 19, 20]. In patients at increased ASCVD risk, higher percent atheroma volume and non-calcified plaque volume have been associated with a stepwise increase of ASCVD events [18, 21]. Higher plaque progression has traditionally been associated with a higher cumulative LDL-C burden and to a lesser extent with other risk factors including hypertension and obesity [22, 23].

In the present study, LDL-C was lower in individuals with diabetes compared with non-diabetic controls, which coincided with the higher use of statins in diabetic patients. As expected, body mass index and systolic blood pressure were higher in the diabetes group. Following adjustment for a wide range of cardiovascular risk factors, comprising body mass index, systolic blood pressure, LDL-C, triglycerides, lipoprotein(a), smoking, family history of CAD and statin treatment, the association between diabetes and plaque progression persisted. In a prior study in patients with stable CAD, the combination of hyperglycemia and low HDL-C was also independently associated with rapid plaque progression [7]. Collectively, these findings imply a direct impact of diabetes on plaque progression independent of other ASCVD risk factors, which is in line with preclinical data showing that hyperglycemia hinders the anti-atherogenic properties of lipid lowering interventions [24].

### Adverse plaque phenotypes and progression

While having a comparable prevalence of adverse plaque features at baseline, diabetic individuals showed a significantly higher prevalence of high-risk plaque and low-density plaque after 10 years of follow-up. Recent post-hoc analyses from the Scottish Computed Tomography of the Heart (SCOT-HEART) trial underscored the prognostic significance of high-risk plaque features and in particular of low-density plaque on cardiovascular event rates during follow-up. In subanalyses, the authors showed that low-attenuation, non-calcified plaque burden (referred to as “low-density plaque” in this study) surpassed traditional risk scores, coronary artery calcium scoring and stenosis severity as the strongest predictor of acute coronary syndromes [25]. One of the factors contributing to plaque progression and adverse plaque features is a chronic pro-inflammatory state [26, 27]. Indeed, diabetes has been characterized as a chronic inflammatory disease with enhanced inflammation of the vascular wall, as substantiated in PET/CT studies [28, 29]. A direct relation between hyperglycemia and vascular inflammation has been attributed to a wide variety of pro-inflammatory effects: the presence of pro-inflammatory mononuclear cells in the bloodstream with an increased propensity towards trans-endothelial migration, a glucose-elicited increase in oxygen radical formation, mitochondrial dysfunction and increased formation of advanced glycation end-products promoting endothelial dysfunction [30, 31]. Collectively, this implies that diabetes is associated with accelerated atherosclerosis progression despite adequate control of traditional cardiovascular risk factors, which may be in part be due to a persistent pro-inflammatory state accelerating atherogenesis. Dedicated studies using various targets in this inflammatory cascade are currently ongoing [32]. These studies will elucidate whether anti-inflammatory strategies on top of optimal cardiovascular prevention regimens will be able to reduce accelerated plaque formation in diabetic patients.

### Clinical implications

The observation that diabetic patients are characterized by accelerated plaque progression and a higher prevalence of adverse plaque features implies a markedly increased ASCVD risk despite adequately controlled traditional cardiovascular risk factors. These data concur with historical landmark cardiovascular outcome trials, showing a higher residual ASCVD risk in diabetic patients in the active treatment arm even when compared with the placebo group in non-diabetic patients [33]. This large residual ASCVD risk in diabetic patients underscores the need for intensified cardiovascular risk management strategies with a special focus on inflammation. Previous trials have demonstrated promising results in attenuating coronary plaque progression using evolocumab and icosapent ethyl [34–36]. In support, the Colchicine Cardiovascular Outcomes Trial (COLCOT) reported a 35% reduction of ischemic cardiovascular events following treatment with low-dose colchicine versus placebo in the diabetic subgroup, compared with a 23% reduction in the original study population [37]. Recent studies in type 2 diabetes also reported regression of coronary non-calcified plaque volume following Sodium-Glucose Transport Protein 2 inhibitor (SGLT2i) therapy and improved coronary plaque stabilization following treatment with both SGLT2i and glucagon-like peptide-1 receptor agonists (GLP1-RA) [38–40]. Whether these beneficial effects reflect anti-inflammatory effects of SGLT2i and GLP1-RA remains to be established.

### Study limitations

This study has several limitations. First, it was a single-center study with a relatively limited sample size. The limited number of participants with diabetes may have limited the power of the present study. Diabetes was defined by medical history and self-report, the presence of subclinical diabetes in the patients without diabetes cannot be excluded and may have led to an underestimation of the effect size. Second, the study was underpowered to detect pericoronary adipose tissue (PCAT)-related inflammatory effects. Several larger studies have investigated this pathway: Overgaard et al. demonstrated that baseline PCAT attenuation was associated with increased total and non-calcified plaque volumes during a follow-up of 12 months [41]. Another study showed that PCAT attenuation was higher in patients with poorly controlled type 2 diabetes compared to non-diabetic patients [42]. This represents an important area for future investigation in larger diabetes cohorts with longer-term follow-up. Third, absence of repetitive HbA1c testing during the follow-up period, precluding detailed insight into the degree of glucose regulation. However, at baseline average glycemic control was close to the recommended target of 53 mmol/mol. Fourth, the difference in CCTA scanners and protocols between baseline and follow-up may have influenced the findings. Despite adjustment for scanner type and settings in the plaque analysis, plaque composition might have been interpreted differently.

## Conclusions

In this 10-year follow-up study using serial CCTA imaging, we show that type 2 diabetes is associated with a threefold increase of coronary plaque progression as well as increased development of high-risk plaque and low-density plaque, independent of traditional ASCVD risk factors and baseline plaque volumes. These data imply that beyond strict regulation of traditional risk factors, novel strategies with alternative targets such as inflammation might benefit especially patients with type 2 diabetes.

## Supplementary Information

Below is the link to the electronic supplementary material.

Supplementary Material 1

## Data Availability

The datasets used and/or analyzed during the current study are available from the corresponding author on reasonable request.

## Abbreviations

CCTA: Coronary computed tomography angiography
PAV: Percent atheroma volume
ASCVD: Atherosclerotic cardiovascular disease
HDL-C: High density lipoprotein cholesterol
CAD: Coronary artery disease
AI: Artificial intelligence
CACS: Coronary artery calcium scoring
PCI: Percutaneous coronary intervention
AI-QCT: Atherosclerosis imaging quantitative computed tomography
CPV: Percent calcified plaque volume
NCPV: Percent non-calcified plaque volume
HU: Hounsfield unit
LDL-C: Low-Density Lipoprotein Cholesterol
Lp(a): Lipoprotein(a)
IQR: Interquartile Range
BMI: Body Mass Index
CAD-RADS: Coronary Artery Disease: Reporting and Data System
SGLT2i: Sodium-Glucose Transport Protein 2 Inhibition
GLP-1 RA: Glucagon-Like Peptide-1 Receptor Agonist
PCAT: Pericoronary adipose tissue
