# Supplementary material for: Increased high-risk plaque burden in type 2 diabetes: a 10-year follow-up study
Source: Cardiovasc Diabetol. 2025 Nov 5;24:423. doi: 10.1186/s12933-025-02977-1 (PMC12590748; doi:10.1186/s12933-025-02977-1)
Supplement: Supplementary file 1 — Supplementary Material 1 [file 12933_2025_2977_MOESM1_ESM.docx]

**Increased high-risk plaque burden in type 2 diabetes: a 10-year follow-up study**

Emilie L. Gaillard, Sophie H.M. Cramer, Nordin M.J. Hanssen, Michiel J. Bom, Steven A. J. Chamuleau, R. Nils Planken, Andrew D. Choi, S. Matthijs Boekholdt, Erik S.G. Stroes, Paul Knaapen, and Nick S. Nurmohamed

**Supplemental Material**

**Supplementary Table 1. Cardiovascular Risk Factor Control at Baseline in Patients With and Without Type 2 Diabetes**

| **Characteristic** | **Overall**,  N = 267 | **Diabetes**,  N = 44 | **No Diabetes**, N = 223 | **p-value** |
| --- | --- | --- | --- | --- |
| LDL-c at 2008 target (<2.6 mmol/L with CAD, <3.4 mmol/L without CAD) | 189 (71%) | 28 (64%) | 161 (72%) | 0.3 |
| Blood pressure at target (<140 mmHg) | 141 (53%) | 19 (43%) | 122 (55%) | 0.2 |
| BMI at target (18.5-25 kg/m²) | 90 (34%) | 4 (9%) | 86 (39%) | <0.001 |
| Not smoking | 187 (70%) | 35 (80%) | 152 (68%) | 0.13 |

Data are presented as n (%)

**Supplementary Table 2. Coronary Plaque Characteristics at Baseline and Follow-up**

| **Characteristic** | **Diabetes (n=44)** | **No Diabetes (n=223)** | **p-value** |
| --- | --- | --- | --- |
| **Percent Atheroma Volume (%)** |  |  |  |
| Baseline | 5.1 (1.7, 10.9) | 2.2 (0.5, 5.8) | 0.002 |
| Follow-up | 13.1 (6.4, 21.8) | 3.8 (1.2, 10.8) | <0.001 |
| Change | 6.0 (1.5, 11.7) | 1.6 (0.4, 4.8) | <0.001 |
| **Percent Calcified Plaque Volume (%)** |  |  |  |
| Baseline | 1.1 (0.0, 4.4) | 0.1 (0.0, 1.6) | 0.003 |
| Follow-up | 6.3 (1.2, 11.5) | 1.3 (0.0, 4.3) | <0.001 |
| Change | 3.3 (0.6, 7.5) | 0.7 (0.0, 2.4) | <0.001 |
| **Percent Non-Calcified Plaque Volume (%)** |  |  |  |
| Baseline | 3.7 (1.4, 7.6) | 1.7 (0.5, 4.0) | 0.002 |
| Follow-up | 6.9 (3.7, 10.9) | 2.5 (0.9, 5.7) | <0.001 |
| Change | 2.4 (0.6, 4.0) | 0.5 (0.0, 2.1) | <0.001 |
| **Low-Density Plaque** |  |  |  |
| Baseline | 6 (14%) | 34 (15%) | 0.786 |
| Follow-up | 17 (39%) | 39 (17%) | 0.002 |
| **High-Risk Plaque** |  |  |  |
| Baseline | 19 (43%) | 67 (30%) | 0.089 |
| Follow-up | 28 (64%) | 81 (36%) | <0.001 |

Data are presented as median (quartile 1, quartile 3) or n (%).

**Supplementary Table 3. Association Between Type 2 Diabetes and Coronary Plaque Presence**

| **Outcome** | **Model** | **OR (95% CI)** | **p-value** |
| --- | --- | --- | --- |
| Presence of High Risk Plaque at baseline | 1 | 1.83 (0.93-3.60) | 0.083 |
|  | 2 | 1.26 (0.58-2.70) | 0.560 |
|  | 3 | 0.99 (0.39-2.52) | 0.987 |
| Presence of High-Risk Plaque at follow-up | 1 | 2.75 (1.38-5.48) | **0.004** |
|  | 2 | 2.33 (1.11-4.88) | **0.025** |
|  | 3 | 2.44 (1.07-5.55) | **0.034** |
| Presence of Low-Density Plaque at baseline | 1 | 1.78 (0.87-3.63) | 0.115 |
|  | 2 | 1.11 (0.48-2.55) | 0.802 |
|  | 3 | 0.61 (0.23-1.65) | 0.329 |
| Presence of Low-Density Plaque at follow-up | 1 | 2.88 (1.45-5.70) | **0.002** |
|  | 2 | 2.50 (1.21-5.17) | **0.014** |
|  | 3 | 2.56 (1.10-5.96) | **0.029** |

Adjusted odds ratios from logistic regression models for adverse coronary plaque development (defined as presence of at least one high-risk or low-density plaque) in patients with type 2 diabetes. Model 1: Unadjusted analysis. Model 2: Adjusted for baseline percent atheroma volume. Model 3: Adjusted for baseline percent atheroma volume, demographics (sex, age) and cardiovascular risk factors (BMI, systolic blood pressure, LDL cholesterol, lipoprotein(a), triglycerides, hypertension, current smoking, family history of premature coronary artery disease) and statin use.
